# The subunit composition of the mammalian Mediator complex is not conserved in all vertebrates: Insights from evolutionary plasticity of fish genomes

**DOI:** 10.1002/pro.70566

**Published:** 2026-04-11

**Authors:** Radka Symonová, Thomas J. Near, Jan Kubečka

**Affiliations:** ^1^ Faculty of Science University of South Bohemia České Budějovice Czech Republic; ^2^ Institute of Hydrobiology Biology Centre of the Czech Academy of Sciences České Budějovice Czech Republic; ^3^ Department of Ecology and Evolutionary Biology, Osborn Memorial Labs Yale University New Haven Connecticut USA; ^4^ Yale Peabody Museum Yale University New Haven Connecticut USA

**Keywords:** fish‐specific Mediator paralogs, med13b, med31l, teleost genome duplication

## Abstract

The Mediator complex is an indispensable, multi‐subunit protein transcriptional coactivator with a central role in gene expression in Eukaryotes. Among vertebrates, its molecular structure and subunit composition are well‐known only for mammals. Genes encoding fish Mediator subunits remain unknown even for zebrafish that is otherwise the best explored fish species. Here, we first reconstructed genes encoding Mediator subunits from 12 brain transcriptomes of a percid fish, pikeperch (*Sander lucioperca*). These data revealed two additional fish‐specific paralogous genes (*med13b* and *med31l*) and a missing paralog (*med12l*) in comparison to genes of mammalian Mediator subunits. The Med13 subunit is encoded by two paralogs in basal (coelacanth) as well as derived sarcopterygians (mammals), while three paralogs are present in numerous but not all fish lineages. All three *med13* paralogs were highly transcribed in the juvenile pikeperch brain. Our molecular‐phylogenetic analysis of the three fish *med13* paralogs revealed the evolutionary origin of the additional *med13b* paralog from the teleost‐specific genome duplication. The additional paralog *med31l* encoding the Med31 subunit shows a more limited occurrence among fishes with potentially different ways and times of its origin—tandem duplication on the same chromosome, translocation in the opposite strand of another chromosome or a consequence of whole‐genome duplication. The mammalian Mediator complex is not universal to all vertebrates and paralogs encoding subunits of the Mediator complex are not conserved across all vertebrates. Further research is needed to explore fish‐specific genes encoding subunits of the Mediator complex and their tissue‐specific transcription.

## 1 INTRODUCTION

**FIGURE 1:**
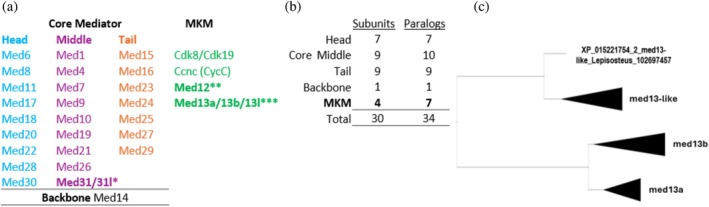
Overview of pikeperch genes and their paralogs encoding Mediator complex subunits identified also in other fishes. (a) Schematic representations of the pikeperch brain Mediator complex with modules, Mediator subunits and the corresponding genes and their paralogs (according to Li et al., 2024). (b) Count summary of pikeperch Mediator subunits and their paralogs. (c) Evolutionary relationships between paralogs of subunit Med13 visualized as an auto‐collapsed tree topology of the med13 paralogs in fish species with all three paralogs. Based on amino acid sequences of protein products of each paralog. A more detailed tree is available in Figure S1 with the underlying FASTA sequences in Table S2. Cdk8/19 kinase module; Ccnc, cyclin C; MKM, mediator kinase module. *Two paralogs described as *med31* and gene IDs LOC116054995 and *med31*. **Missing vertebrate paralog *med12*. ***Three different paralog, all with a high transcription rate in comparison to other transcripts.

The eukaryotic Mediator complex is a highly conserved protein assembly comprising a large Core, the proper Mediator complex, and a dissociable Cdk8/Cdk19 kinase module (CKM, sensu Lambert et al., 2023) also known as Mediator kinase module (MKM, sensu Richter et al., 2022). The Core is further subdivided into Head, Middle, Backbone, and Tail that in mammals consists of the lower and upper tail. These modules are formed by Mediator protein subunits Med1, Med4, Med6—Med31, Cdk8/Cdk19, and CycC encoded by genes of the same names (Figure 1a). Three of the four MKM subunits are each encoded by two mostly mutually exclusive paralogs *med12/12L*, *med13/13L*, *cdk8/cdk19* in vertebrates (Luyties & Taatjes, 2022). Presence of these paralogs is specific to metazoans in comparison to the well‐explored yeast Mediator (Lambert et al., 2023). The Mediator molecular structure and subunit composition are known from yeast and other fungi (Lambert et al., 2023), plants (Samanta & Thakur, 2015; Yang et al., 2016), fruit fly (Park et al., 2001), and mammals (Richter et al., 2022), yet they remain unknown for lower vertebrates including fish. Thus, the mammalian Mediator complex currently represents all vertebrates, and its Core includes 26 Med subunits (Chen et al., 2021; Rengachari et al., 2021; Richter et al., 2022). The subunit composition is conserved in mammals, whereas the DNA sequence of the subunits is not conserved at all (Richter et al., 2022). Functionally, the Mediator initiates gene transcription by bridging transcription factors (TF) bound to enhancers with RNA polymerase II (RNAPII) transcription machinery at promoters. It functions as a critical coregulator during gene transcription, whereby the Core recruits RNAPII and facilitates the assembly of the pre‐initiation complex at promoters. In contrast, MKM prevents RNAPII binding to the Core while exerting a positive or negative influence on gene transcription (Li et al., 2024).

Several studies investigated specific genes and/or individual Mediator subunits in fish—five of them explored the subunit Med12 in the neurodevelopmental context (Hong et al., 2005; Keightley et al., 2011; Rau et al., 2006; Shin et al., 2008; Wang et al., 2006) and two the subunit Med27 in the context of a neurodevelopmental disease (Dürr et al., 2006; Li et al., 2025). The latter identified 12 TFs as direct targets of the Med27 subunit ( *neurod1* , *egr4*, *mxd3*, *gbx1* , *rorab*, foxo3a, *fosab* , *thap4*, *nhlh2*, *nrf1*, *en2a* , and *zbtb12.2*), four of them (underlined) as critical for zebrafish neurodevelopment. A study focused on conserved intrinsically disordered regions of Mediator subunits included merely a single fish species, the zebrafish, along with other 146 eukaryotes (Nagulapalli et al., 2016). This study did not investigate all Mediator subunits in zebrafish. Moreover, zebrafish is not the best suitable and representative fish species regarding its peculiar genome composition and a biased transposon composition (Matoulek et al., 2023; Vohnoutová et al., 2023). Finally, the same group produced a database of Mediator proteins, MedProDB, with several fish species listed (Bhardwaj et al., 2021). However, their records of fish Mediator subunits are incomplete, omitting paralogs and protein isoforms and miss species shown here (below) with additional paralogs. The main objective of this study is to reconstruct genes and their paralogs encoding Mediator subunits in fish species with available reference genome and to fill this basic knowledge gap.

## 2 DISCUSSION

Using our transcriptomics data (Symonová, Jůza, Tesfaye, Brabec, Bartoň, et al., 2025a; Symonová, Jůza, Tesfaye, Brabec, Sajdlová, & Kubečka, 2025b), we reconstructed genes encoding subunits of the Mediator complex transcribed in the juvenile pikeperch (*Sander* [*Stizostedion*] *lucioperca*) brain. This data allowed for identification and quantification of transcript levels (Table S1) of genes encoding 27 Core Mediator and Backbone subunits and four subunits of the MKM encoded by seven paralogs (Figure 1a,b). All genes were validated in the NCBI/Gene and NCBI/Nucleotide database (Sayers et al., 2025) and their sequences were analyzed by basic local alignment search tool (Altschul et al., 1990). There are following differences to the currently known mammalian Mediator complex in pikeperch: (i) the MKM Med13 subunit was encoded by three distinct paralogs (*med13a*, *13b*, *13L*), a situation so far unknown for mammals whose MKM contains only two paralogs of Med13 (*MED13* and *MED13L*; Richter et al., 2022; Lambert et al., 2023); (ii) on the other hand, only a single paralog of the Med12 subunit of the MKM was revealed in pikeperch, that is, the *MED12L* paralog known for mammals is missing; and (iii) the Core Med31 subunit was represented by two distinct paralogs in pikeperch (*med31* and *med31l*; Figure 1c), again a situation so far unknown for mammals, where a single *Med31* gene has been reported (e.g., Lambert et al., 2023).

### 2.1 Genes of the fish Med13 subunit

**FIGURE 2:**
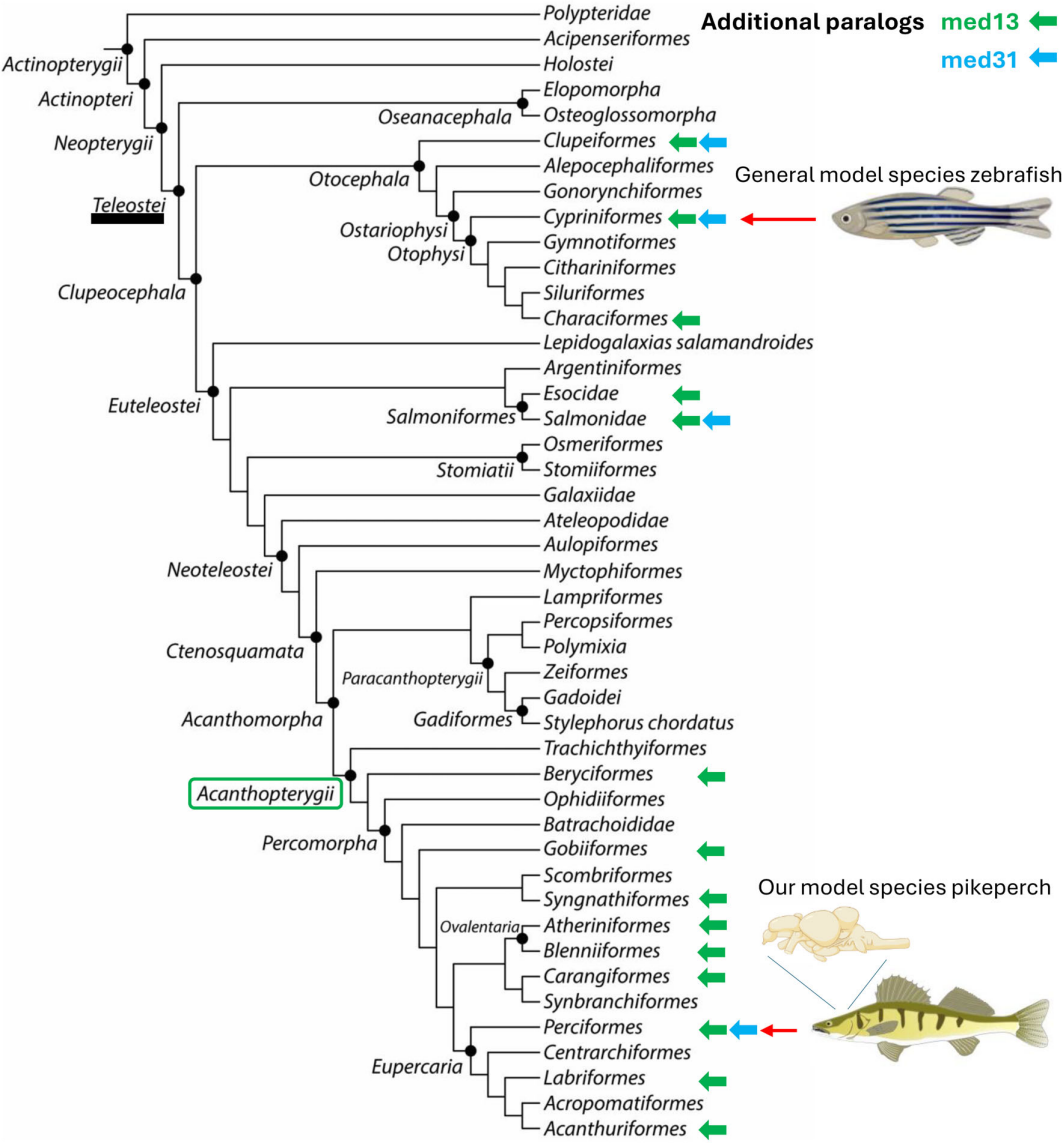
Distribution of additional paralogs med13 and med31 across fish phylogeny. Based on Near & Thacker, 2024.

The pikeperch subunit Med13 of the MKM is encoded by three gene paralogs *med13a* (LOC 116058278), *med13‐like* (LOC116047565), and another one equally annotated *med13‐like* (LOC116034974). An additional fish‐specific paralog *med13‐like* was recorded in 61 fish species with a reference genome assembly available in the NCBI database across fish phylogeny (Figure 2). Among teleosts, the additional *med13‐like* paralog along with the *med13a* paralog was recorded in the basal teleost lineages (Clupeiformes, Cypriniformes, Characiformes, and Salmoniformes) and then in the most derived and diverged group Acanthopterygii (Figure 2; Near & Thacker, 2024). To resolve the origin of the two *med13‐like* paralogs, we performed a molecular‐phylogenetic analysis of protein products of all three co‐occurring teleost *med13* paralogs using the online tool multiple alignment using fast fourier transform (MAFFT) in its default setting (mafft ‐‐thread 8 ‐‐threadtb 5 ‐‐threadit 0 ‐‐reorder ‐‐auto input > output) (Katoh et al., 2019). This analysis yielded a clustering pattern for one of the teleost *med13l* paralogs as the basal and corresponding to the sarcopterygian MED13L. The *med13a* paralog was sister to another *med13l* and both appear as products of the teleost‐specific whole‐genome duplication (TSGD; Glasauer & Neuhauss, 2014). This suggests that the additional *med13l* (116047565) paralog in pikeperch should be annotated as *med13b* (Figures 1c and S1, Table S2) due to its potential origin as a product of the TSGD. The *med13b* paralog was, however, not retained across all teleost lineages during the subsequent process of lineage‐specific rediploidization which reflects on its current distribution across the fish phylogeny (Robertson et al., 2017). The gene length of the med13 paralogs was between ca. 25,000 and 220,000 nt and the exon count was in the range 29–35 with 2045–2178 amino acids encoded. Transcripts of all these three med13 paralogs were highly abundant in the juvenile pikeperch brain (Table S1), providing evidence of their biological activity. To elucidate any potential functional differences between proteins encoded by the three med13 paralogs in pikeperch brain, we performed an in silico analysis of physical and chemical traits of their amino acid sequences using an online tool ProtParam of the Expasy tool collection (Duvaud et al., 2021). This analysis has not revealed any difference between protein products of the three med13 paralogs and the results are summarized in Table S3.

In black rockcod (*Notothenia coriiceps*), even five *med13‐like* paralogs were identified (LOC104956413, LOC104941230, LOC104957374, LOC104962796, and LOC104957379 located on four different unplaced scaffolds) without any *med13a* being annotated. However, three of the five paralogs have substantially fewer exons (2, 11, and 15), encode fewer amino acids (108, 702, and 1185), and hence can be considered unfunctional. The situation in zebrafish illustrates the nomenclature inconsistency in fish—two paralogs, *med13a* and *med13b*, are known in zebrafish, although the *med13l* paralog was recorded in two other species belonging to the order Cypriniformes as zebrafish does. Moreover, the protein product of the zebrafish gene *med13b* (GeneID:564707) is annotated as XP_693130 “mediator of RNA polymerase II transcription subunit 13‐like” referring to the nomenclature known in other teleost species. Indeed, at the amino acid sequence level, the protein product of the zebrafish gene *med13b* clusters with *med13l* of other species, whereas the product of the zebrafish *med13a* gene (GeneID:100004024) clusters with proteins annotated as med13a. This indicates that zebrafish *med13* paralogs should have been annotated as med13a and med13l.

Based on the exon and amino acid counts, all three *med13* paralogs in other teleost species appear to be functional as in the pikeperch brain. The above‐described retention of the additional fish‐specific med13 paralog may reflect the high importance of the MED13 protein subunit known for mammals in zygotic genome activation, growth and development (Miao et al., 2018; Villeret et al., 2024). Mammalian *Med13* genes usually have 30–33 exons and code for ca. 2174 amino acids (NCBI).

### 2.2 Med31 subunit

The two paralogs of the Med31 subunit transcribed in the pikeperch brain were annotated as *med31* and as LOC116054995 and both described as “mediator of RNA polymerase II transcription subunit 31.” One of these paralogs can be considered a fish‐specific *med31l*, however, it is not present in all teleost species. Beside pikeperch, we identified an additional paralog in further six teleost species (five genera) of the 241 listed in National Centre for Biotechnology (NCBI) having a reference genome (Figure S2). Two species belong to the order Perciformes, three species in two genera belong to the order Clupeiformes, and a single species belongs to Salmoniformes and Cypriniformes, respectively. Moreover, genes of the *med31l* with such a limited and patchy distribution had obviously not the same origin and consequently they differ in timing of their origin among the six species: (1) a tandem duplication was the most likely mechanism of its origin in pikeperch (Perciformes) and carp (*Cyprinus carpio*, Cypriniformes), since both genes are located on the same chromosome and in the same direction although in a different distance from each other (Figure S3); (2) a translocation into the opposite direction of another chromosome might have occurred in the American shad (*Alosa sapidissima*, LOC121723742 on chromosome 1, LOC121708973—the potential *med31l* on chromosome 5), allis shad (*Alosa alosa*, LOC125299835 on chromosome 1, the *med31l* paralog LOC125291442 on chromosome 2) and the Atlantic herring (*Clupea harengus*, med31/105894264 on chromosome 8, LOC105899203 on chromosome 22—the potential *med31l*) (all three Clupeaformis); (3) *med31l* can be considered a consequence of a lineage‐specific whole‐genome duplication (WGD) in lake whitefish (LOC121573610 on chromosome 9, LOC121579809 on chromosome 13) (*Coregonus clupeaformis*, Salmoniformes, Macqueen & Johnston, 2014); finally, (4) the combination of another lineage‐specific WGD (David et al., 2003) and the tandem duplication can be found in carp, where even two additional *med31l* paralogs, that is, altogether three med31 genes (LOC109064444 unplaced with four exons, LOC122141335 and LOC122141204 both on chromosome B21 with four exons—the potential *med31l*) were identified. There is no genome assembled to the chromosome level for the sixth species with two med31 paralogs, emerald rockcod (*Trematomus bernacchii*, Perciformes), hence, its location on specific chromosome(s) remains unknown. This species has both med31 genes (LOC117469549, LOC117469495) in the complement direction of a single unplaced scaffold NW_022987876 of the reference genome fTreBer1.1 (NCBI) which is similar to the situation in pikeperch (also Perciformes).

The genes coding for the Med31 subunits were highly variable in their size among species but mostly similar within the species: pikeperch 6655 and 6533 nt, the American shad 3121 and 3574 nt, carp 1414‐175 nt, the Atlantic herring 2591 and 2856 nt, emerald rockcod 7355 and 7488 nt. Only in lake whitefish, one gene had 7968 nt while the other one 21,964 nt. Exon counts were in the range between one (carp) and four (most of the species and their genes). Despite the differences in gene length and exon counts, these genes encode between 128 and 145 amino acids. The gene encoding the mammalian MED31 subunit has also four exons with 131 amino acids coded (NCBI), although there are several exceptions in the amino acids count (NCBI).

The above‐mentioned traits of the additional fish‐specific paralogs of Mediator subunits indicate that there were different mechanisms of their origin and a different timing of origin between the two *med31l* and *med13l* paralogs.

For the MKM, both paralogs *cdk8* and *cdk19*, currently known in the mammalian Mediator complex, were transcribed in the juvenile pikeperch brain. However, *cdk8* transcripts showed about twofold higher abundance compared to *cdk19* (Table S1). The *cdk8* paralog was found in 260 fish species and *cdk19* paralog was found in 224 fish species with a reference genome on NCBI. On the other hand, the *med12l* paralog of the MKM module was missing in all teleost fishes while found only in two sharks and a skate, in lungfish, and in coelacanth. This paralog thus was present in early vertebrates and although retained in mammals (i.e., Sarcopterygians) it was lost in teleosts and in majority of ray‐finned fishes. The highest transcription among the Mediator genes in pikeperch brain was recorded for subunits Med8 and Med12 (Table S1). The high transcription of the latter one could potentially compensate for the missing *med12l* paralog.

The retention of the additional paralogs and their novel acquisition in the order Perciformes is kind of surprising because this derived fish lineage is rather known for genome size compaction (average cytological genome size 0.94; Gregory, 2025). Hence, the importance of these additional paralogs must have been high for fish.

There are several reasons why it is important to know the Mediator subunit composition in fish: (1) Fish are crucial models for human developmental disorders, where numerous Mediator subunits play key roles (da Silva et al., 2025). (2) Evolutionary insights into the complexity of Mediator subunits should not be neglected across vertebrates as it has been so far (as shown here). (3) Since the Mediator complex is involved in developmental regulations, knowledge of its interactions with specific TFs in juvenile fish, particularly in the brain, will be crucial to move our knowledge forward (Symonová, Jůza, Tesfaye, Brabec, Bartoň, et al., 2025a; Symonová, Jůza, Tesfaye, Brabec, Sajdlová, & Kubečka, 2025b). Hence, the exact molecular structure and subunit composition of the Mediator and its interactions with general and specific TFs will be fundamental for our future research. 4. Finally, the genomic resources for fish and fish‐like organisms still heavily suffer from inconsistencies in gene annotations (this study) that make their analysis far less straightforward and less effective than for mammals.

The potential roles of Mediator subunits with the additional paralogs can be derived from resources obtained for mammalian model/humans. The three MKM subunits exists in two paralogs in mammals—CDK8/CDK19, MED12/MED12L, and MED13/MED13L (Lambert et al., 2023; Maalouf et al., 2024; Yin & Wang, 2014). The Med13 subunit may be involved in the reversible association of MKM with the Core and hence crucial for the regulation of transcription (Jeronimo et al., 2016; Petrenko et al., 2016). The availability of the extra paralogs in pikeperch can be related to its distinct physiological roles known for humans. CDK19 and MED13L may manifest in tissue‐specific ways (Poss et al., 2013)—CDK8 is ubiquitous across human tissues, CDK19 shows tissue‐specific expression (Tsutsui et al., 2011). A clinical study indicates the importance of CDK19 in neurodevelopment since its disruption causes neurological disorders (Mukhopadhyay et al., 2010). Similarly, MED13L is potentially involved in brain development (Muncke et al., 2003). These findings, although originating from humans, are relevant for fish neurodevelopment regarding the high functional conservation of the Mediator complex across Eukaryotes (Lambert et al., 2023; Poss et al., 2013). Both *cdk8* and *cdk19* paralogs in zebrafish were predicted to enable protein serine/threonine kinase activity and to act upstream of or within protein phosphorylation and none of them is known to be transcribed in central nervous system (Bradford et al., 2022). Hence, the potential contribution of both paralogs to the MKM cannot be excluded.

Our results demonstrate that the subunit composition of the Mediator complex known in mammals is not conserved across all vertebrates. In contrast to mammals, the greater evolutionary dynamics of genes encoding the Mediator subunits shaped the evolution of the Mediator complex in fish. This is in accordance with the fact that fish are the most specious vertebrate lineage (Nelson et al., 2016). Hence, it is necessary to re‐annotate gene names of fish Mediator subunits to remove incongruences like shown above between zebrafish *med13a* and *med13b* and those *med13l* in remaining teleosts. It is anticipated that as the number of species with high‐quality genome assemblies increases, more examples of fish species with additional Med13 paralogs will be identified. The Mediator complex of diverse fish lineages is worthy of more detailed investigations to finally disclose its entire complexity as a consequence of the long evolutionary history of fishes accompanied by the teleost‐specific WGD and multiple lineage‐specific whole‐genome duplications.

## AUTHOR CONTRIBUTIONS

**Jan Kubečka:** Project administration; supervision; resources; writing – review and editing; funding acquisition. **Thomas J. Near:** Visualization; formal analysis. **Radka Symonová:** Conceptualization, data curation, formal analysis, investigation, methodology.

## FUNDING INFORMATION

This study has received funding from project no. R200962402 Fisheries management of a large reservoir under conditions of climate and nutrient change of the Regional cooperation programme of Czech Academy of Sciences and from the ELIXIR CZ Research Infrastructure (IDLM2018131, MEYS CR).

## CONFLICT OF INTEREST STATEMENT

None of the authors have a conflict of interest to disclose.

## Supporting information


**Figure S1.** Gene tree of med13 paralogs based on amino acid sequences.

**Figure S2.** Amino acid sequences of the med31 paralogs, parameters of MAFFT v7 used to produce both gene trees, gene tree of med31 paralogs.

**Figure S3.** Positions of med31 and med13l paralogs in pikeperch and common carp.

**Table S1.** Genes of pikeperch Mediator complex subunits transcribed in two consecutive seasons in brain. The color code of subunit names corresponds to colors used in Figure 1. Transcripts of the additional paralogs med31 and med13 are bold as well as the single copy of med12. The red color means the high(est) value of transcription, the green color means the low(est) value of transcription.

**Table S2.** FASTA sequences of Med13 subunit paralogs.

**Table S3.** Results of the protein parameters analysis with ProtParam (Expasy).

## Data Availability

The data that supports the findings of this study are available in the supplementary material of this article.
