# Supplementary figures and images for: The subunit composition of the mammalian Mediator complex is not conserved in all vertebrates: Insights from evolutionary plasticity of fish genomes
Source: Protein Sci. 2026 Apr 11;35(5):e70566. doi: 10.1002/pro.70566 (PMC13069492; doi:10.1002/pro.70566)

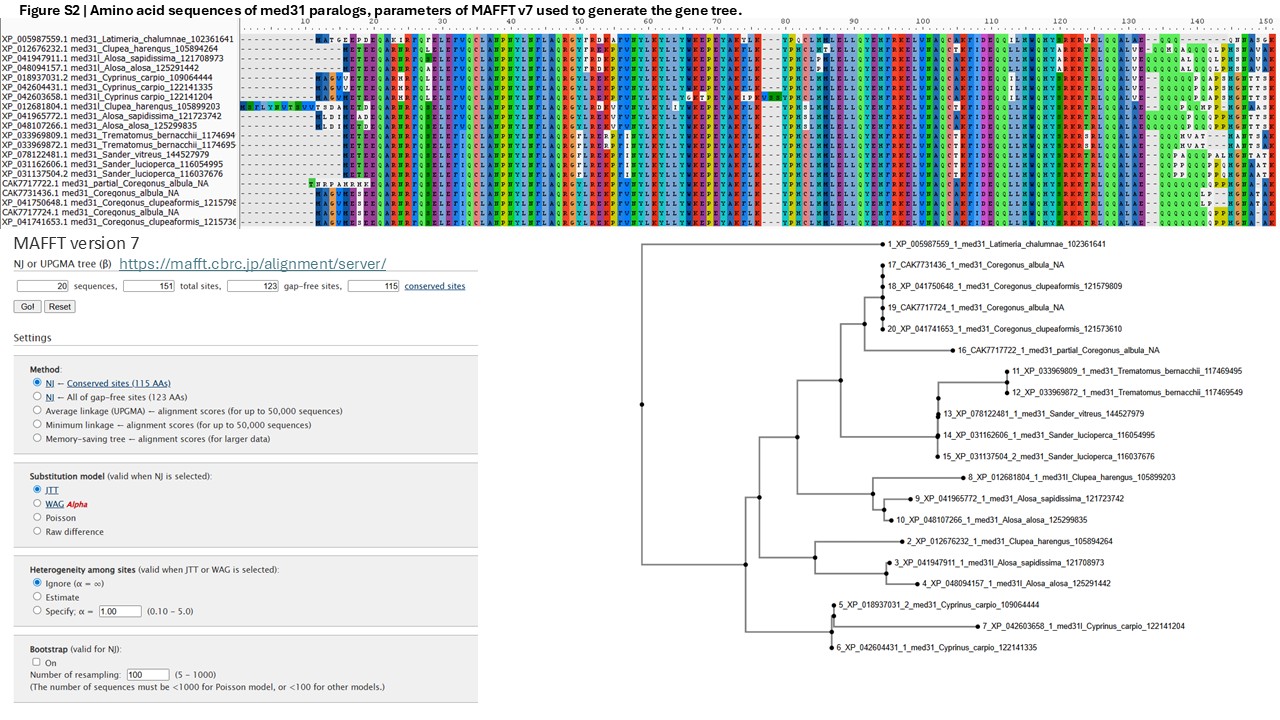

Supplement: Supplementary file 2 — Figure S2. Amino acid sequences of the med31 paralogs, parameters of MAFFT v7 used to produce both gene trees, gene tree of med31 paralogs. [file PRO-35-e70566-s006.jpg]

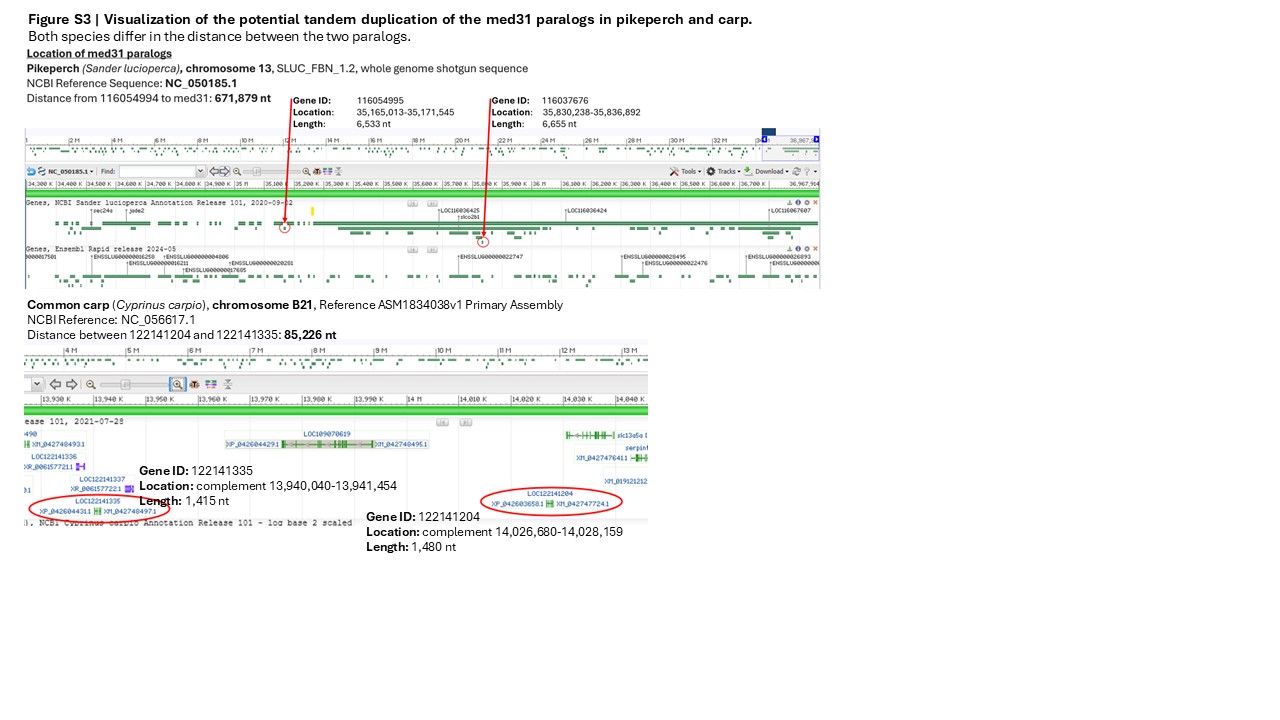

Supplement: Supplementary file 3 — Figure S3. Positions of med31 and med13l paralogs in pikeperch and common carp. [file PRO-35-e70566-s005.jpg]
